# Translation step-cut osteotomy for posttraumatic Cubitus Varus in adults: a retrospective study

**DOI:** 10.1186/s12891-020-03845-7

**Published:** 2020-12-07

**Authors:** Jung Ryul Kim, Yoong Jae Moon, Sung Il Wang

**Affiliations:** 1 Department of Orthopaedics Surgery, Jeonbuk National University Medical School, Research Insitute for Endocrine Sciences and Research Insitute of Clinical Medicine of Jeonbuk National University–Biomedical Research Insitute of Jeonbuk National University Hospital, 567 Baekje-ro, Dukjin-gu, Jeonju, 561-756 Republic of Korea; 2 Department of Biochemistry and Molecular Biology, Jeonbuk National University Medical School, Research Institute of Clinical Medicine of Jeonbuk National University-Biomedical Research Institute of Jeonbuk National University Hospital and Research Institute for Endocrine Sciences, Jeonju, Jeonbuk 54896 Republic of Korea

**Keywords:** Adult, Cubitus varus, Deformity, Elbow, Translation step-cut osteotomy

## Abstract

**Background:**

Cubitus varus is a complex three-dimensional deformity. Various osteotomies have been introduced to correct this complex deformity. The objective of the present study was to evaluate clinical and functional outcomes of adult cubitus varus deformity treated with translation step-cut osteotomy.

**Methods:**

Seventeen consecutive patients with a mean age of 25 years (range, 19–50 years) who underwent translation step-cut osteotomy were enrolled in this study. Their average follow-up period was 28.2 months. Radiographic measurements preoperatively, 3-month postoperatively, and at the last follow-up were compared. Functional outcomes were assessed using Disabilities of the Arm, Shoulder and Hand (DASH), Mayo Elbow Performance Score (MEPS), and Oppenheim criteria.

**Results:**

The mean humerus–elbow–wrist angle improved from 14.7° ± 6.4° (range, 6°–23°) varus preoperatively to 12.1° ± 6.6° (range, 5°–20°) valgus postoperatively (*p* <  0.001). The lateral prominence index improved 9.6% from its preoperative value, showing no significant difference from that of a normal elbow. Osseous union was radiographically demonstrated in 16 patients (except one out of 17 patients) within a mean of 12.7 weeks (range, 8–18 weeks). The motion arc of the elbow at the last follow-up was not significantly (*p* > 0.05) different from that at the initial presentation. Based on Oppenheim criteria, results were excellent for 7, good for 8, and poor for 2 patients. Mean final DASH value and MEPS were 2.5 ± 3.8 points (range, 0–15 points) and 97.0 ± 5.8 points (range, 85–100 points), respectively. With regard to complications, one case had delayed union and one case had transient radial nerve injury.

**Conclusion:**

Translation step-cut osteotomy using Y plate is an efficient procedure to correct varus alignment and flexion-extension deformities so that they are within normal limits of adults with post-traumatic cubitus varus deformity.

**Trial registration:**

Institutional Review Board of Jeonbuk National University Hospital (IRB No. 2020–01-020).

## Background

Cubitus varus is a complex three-dimensional deformity consisting of varus angulation in the coronal plane, internal rotation in the axial plane, and extension in the sagittal plane. It is a frequent complication following treatment of elbow fracture. Although cubitus varus has been conventionally described as a cosmetic deformity with little functional disability, surgical treatment might be necessary when patients are unsatisfied with the appearance of their arms or have late sequelae such as chronic pain, ulnar nerve palsy [1, 2], posterolateral rotary instability [3], and snapping elbow [4]. Various osteotomies have been proposed to correct this complex deformity, including lateral closing wedge, medial opening wedge, dome-shaped, pentalateral, and three-dimensional osteotomies [5–12]. Most of these osteotomies have been performed in young or mixed age groups. However, the clinical course of corrective osteotomy in adults could be different from that seen in growing children as adults have less remodeling capacity compared to younger ones. Adults might be more vulnerable to cosmesis due to lateral protrusion. Lateral protrusion of a distal fragment after a corrective osteotomy might cause a lazy S-shaped deformity [9, 10]. Therefore, surgical correction associated with sufficient medial shift of distal fragment is necessary to prevent S-shaped deformity in adults. Translation step-cut osteotomy is a simple osteotomy that enables three-dimensional correction of coronal, sagittal, and rotational deformities. A triangular wedge-shaped surface created by osteotomy can provide firm stability. Kim et al. [13] have performed this osteotomy for both cubitus varus and valgus deformities with good clinical results. However, adult cubitus varus deformity, particularly for cosmetic correction of lateral prominence, has not been reported yet. We attempted to simultaneously correct not only varus and flexion–extension deformities, but also lateral protrusion of distal fragments with translation step-cut osteotomy in adults. We hypothesized that our step cut osteotomy would be an efficient procedure to correct varus alignment and flexion-extension deformities to be within normal limits.

## Methods

The design and protocol of this retrospective study were approved by the Institutional Review Board of Jeonbuk National University Hospital (IRB No. 2020–01-020). Between October 2006 and April 2014, 17 patients (17 elbows) aged 19 years or older underwent corrective osteotomy for treating cubitus varus. Data regarding their physical and radiographic examinations were reviewed retrospectively using charts and radiographs. These 17 patients were all male patients, with a mean age of 25.7 years (range, 19–50 years) during the osteotomy. Surgical correction was indicated when patients wished to correct the deformity because of an unsightly appearance or impairment to their daily life. No patient had any preoperative problem such as ulnar nerve symptoms or posterolateral instability resulting from deformity. The diagnosis of the initial injury deduced from history and preoperative radiographs was supracondylar fracture in 14 patients, transcondylar fracture of the humerus in one patient, and unknown in two patients. They had been treated with cast immobilization (14 patients) or pinning (3 patients). Their mean age during the initial injury was 8.5 years (range, 4–13 years). The mean interval between injury and surgery was 15.2 years (range, 4–40 years). Their mean follow-up period was 28.2 months (range, 24–55 months).

### Surgical technique

**Fig. 1:**
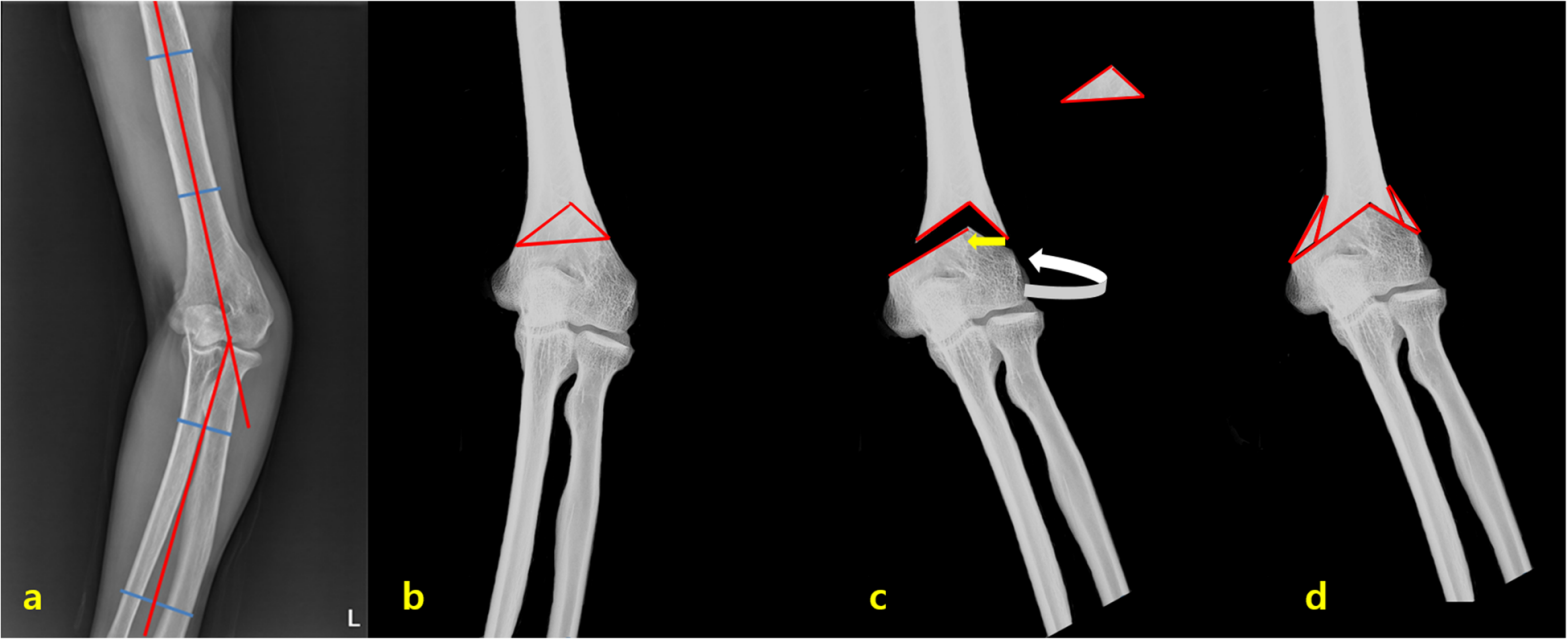
Translation step-cut osteotomy for correction of cubitus varus deformity. **a.** The humerus-elbow-wrist (HEW) angle is measured on an anteroposterior radiograph. **b.** After the correction angle is determined by comparing HEW angles of both elbows, the initial transverse osteotomy line is made about 0.5 to 1 cm superior to the olecranon fossa and perpendicular to the axis of the humerus. The osteotomy is performed along the triangular line. **c.** The deformity is corrected by rotating the distal fragment externally to correction internal rotational (white arrow) and translating it medially to prevent lazy S-shaped deformity (yellow arrow). **d.** After correction, the excised triangular bone is used as supplementary bone graft

Anteroposterior (AP) radiographs of both upper extremities were obtained with the elbow extended and the forearm supinated. The correction value of deformity was determined by comparing humerus-elbow-wrist (HEW) angles of both elbows (Fig. 1a). We constructed our provisional osteotomy after tracing radiographs of the deformed elbow on papers (Fig. 1b, c). We also made an aluminum triangular template of the same shape as paper template obtained in provisional osteotomy for per-operative sterilization and easy use during surgery. The operation was performed with a posterior longitudinal skin incision in supine position. We split the triceps tendon centrally and retracted it on both sides. After placing the triangular template over the proximal portion of the humerus, the outline of the template was marked with a surgical pen (Fig. 1b). Osteotomies were then performed based on the drawn line according to the procedure previously reported by Kim et al. [13]. Deformity was corrected by rotating the distal fragment externally to correction internal and translating it medially to prevent lazy S-shaped deformity (Fig. 1c). After temporary fixation with smooth Steinmann pins, we checked the carrying angle for both elbows. When it was judged that the deformity was properly corrected, we performed the final fixation by applying a single Y plate (Stryker, Selzach, Switzerland) and screws. The excised triangular bone after fixation was used as a supplementary bone graft (Fig. 1d). A removable long-arm splint was applied. Gentle active assistive range of motion (ROM) exercises were started three or four weeks after the surgery. The splint was removed at 6 weeks after the surgery depending on the progress of bony union. All patients were followed up at 2, 6, 12, 18, 24 weeks, and then every 3 months until 1 year after the surgery.

### Radiologic and clinical evaluations

**Fig. 2:**
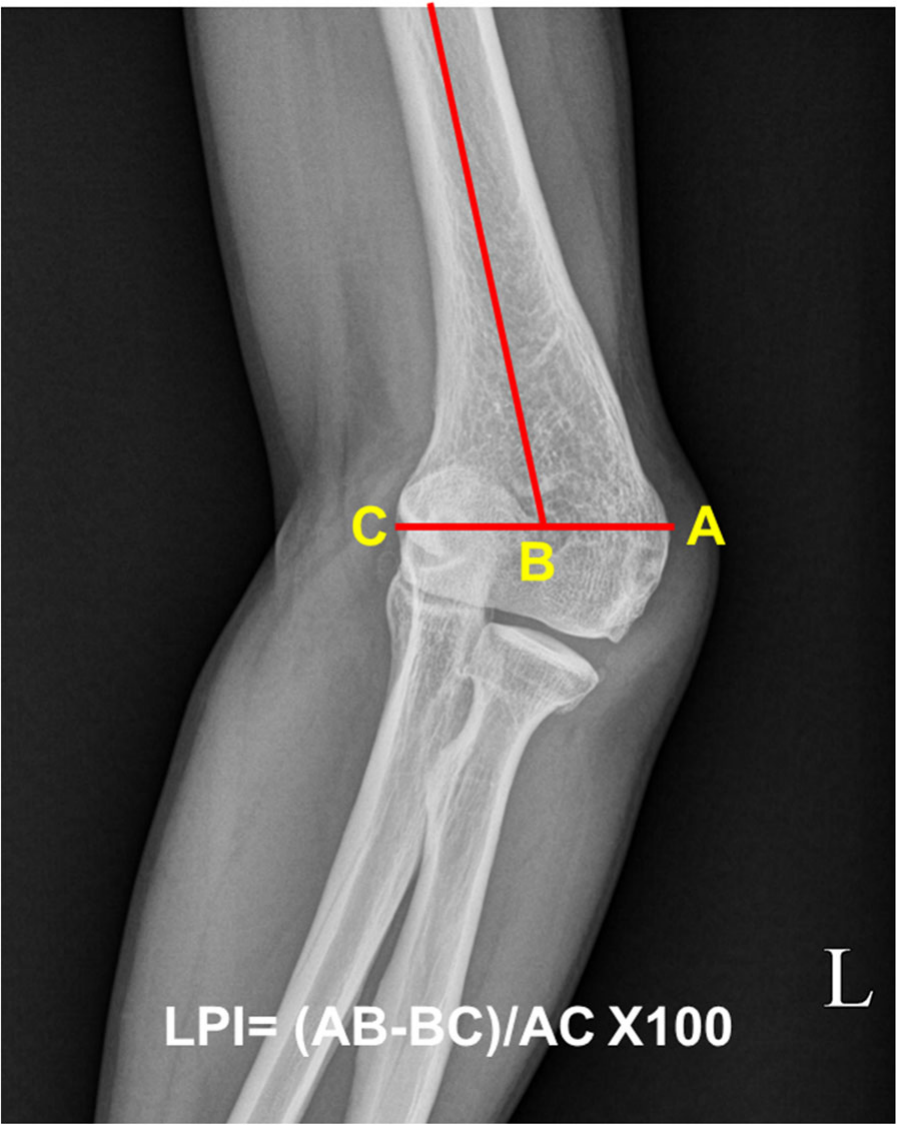
The lateral prominence index (%) is measured on preoperative and postoperative radiographs using the following formula: (AB-BC)/AC × 100. B, cross-link between a line connecting the lateral prominence; A, medial prominence; and C, longitudinal medhumeral axis [16]. This index is usually negative for normal elbows

Radiographic union was determined when callus crossing the osteotomy site was observed in at least 3 cortices AP and lateral elbow radiographs [14]. Delayed union was determined when the radiographic union was not seen even at more than 3 months after the surgery [15]. HEW angle and lateral prominence index (LPI) were evaluated on pre- and post-operative AP radiographs of both elbow joint to assess the correction angle [16] (Fig. 2). Measured values of the deformed elbow were compared with values of the contralateral normal elbow.

Interobserver reliability was determined using intra-class correlation coefficients (ICCs) for three orthopedic surgeons who measured radiographs independently in a blinded fashion. Four weeks after measurements were made by all three surgeons, one surgeon repeated radiographic measurements to assess intra-observer reliability. ICC values of 1 or greater than 0.8 indicated perfect or excellent reliability, respectively (Table 1).

**Table 1:** Interobserver reliability of radiographic measurement

Radiographic measurement	ICC	95% CI
AP humerus- elbow- wrist angle (°)	0.89	0.71–0.96
AP lateral prominence index (%)	0.85	0.63–0.94

*ICC* intraclass correlation coefficient, *95%* confidence interval. The ICCs and their 95% Cis were used to summarize the interobserver reliability of the radiographic measurements and were calculated in the setting using a two-way random effect model assuming a single measurement and absolute agreement

The extent of rotational deformity was determined by physical examination. The angle between the forearm and the back was measured with the elbow in 90° flexion and the shoulder in hyperextension [17]. ROM of the elbow was measured while holding medial and lateral condyles in the same horizontal plane to see the true flexion contracture of the elbow [15]. Results of correction were evaluated based on the criteria of Oppenheim et al. [13, 16]. They are rated as excellent, good, or poor (Table 2). Disabilities of the Arm, Shoulder, and Hand (DASH) and Mayo Elbow Performance Score (MEPS) were used to assess postoperative functional outcomes. We used 11 basic assessment items in DASH. Optional work and sports/performing arts modules were not used. DASH score ranged from 0 to 100, with higher score indicating worse function and lower score indicating better function related to upper extremity disability [18]. MEPS consisted of assessment of pain, arc of motion, stability, and patient rating of daily function. The scale ranged from 0 to 100, with a higher score indicating a better outcome [19].

**Table 2:** Modified criteria of Oppenheim at al

Results	Correction of the HEW angle (°)	Loss of ROM (°)	Complications
Excellent	0–5	0–5	None
Good	6–10	6–10	Scarring or a lazy-S deformity
Poor	> 10	> 10	Other complications (infection, myositis ossificans, and neurovascular injury)

### Statistical analysis

Data are presented as mean ± standard deviation. Differences between pre- and post-operative ranges of motion and radiographic values for each deformity were determined by paired *t*-test. Unpaired *t* test was used to evaluate statistical significance between normal and deformed elbows. All data were analyzed by YJM using SPSS software (version 18.0). A *p* value of less than 0.05 was considered statistically significant.

## Results

### Radiographic results

**Fig. 3:**
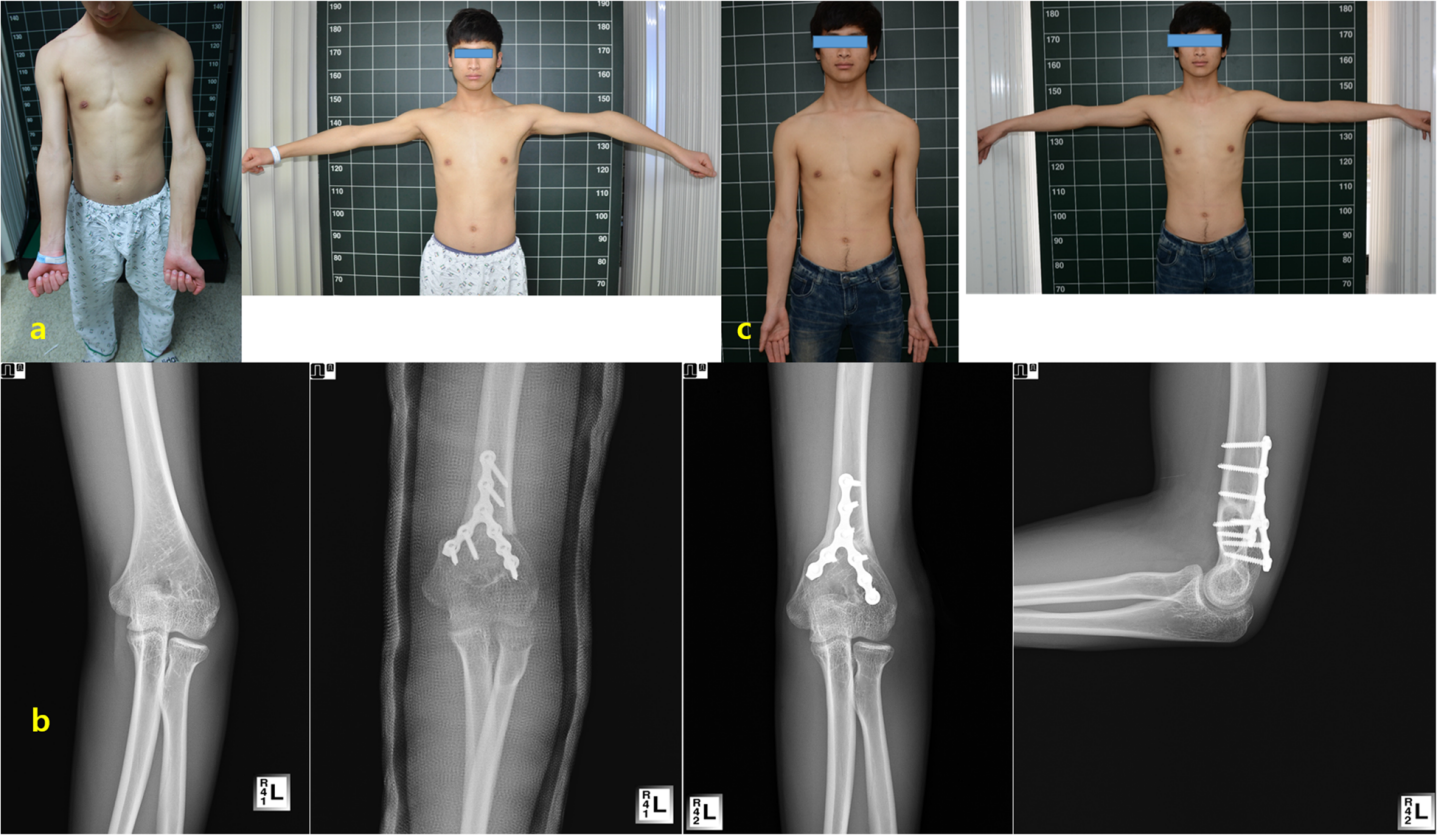
**a** A19-year-old male (Patient 17) who presented with a cubitus varus deformity; **b** Preoperative, postoperative, and last follow radiographs showing translation step-cut osteotomy of deformity with Y plate and screws; **c** At the last follow up, HEW angle and LPI were corrected to valgus 20° and 5%, respectively. He showed excellent results with an angle of elbow motion of 130° (extension, 0°; further flexion, 130°)

All patients except one demonstrated osseous union of the osteotomy site at a mean period of 12.7 ± 3.0 weeks (range, 8–18 weeks) after surgery. HEW angle and LPI radiographic measurements showed excellent interobserver reliability (Table 1). Mean HEW angles of deformed and normal elbows were − 14.7° ± 6.4° preoperatively and 13.6° ± 5.2°, respectively. The mean preoperative varus deformity was 28° ± 6.4° (range, 14°- 48°). The mean HEW angle was 11.5° ± 8.2° at 3 months postoperatively and 12.1° ± 5.2° at the last follow up. Mean LPI values of normal and deformed elbows were − 3.7% ± 5.7% and − 10.3% ± 5.8% preoperatively, respectively. At the final follow-up, the mean LPI of the deformed elbow was − 0.7% ± 4.6%, which was an improvement of 9.6% from its preoperative value (Fig. 3).

Correction of preoperative deformity in HEW angle and LPI radiographic measurements were maintained from 3 months postoperatively to the last follow-up (*p* = 1.000), respectively. In addition, comparison of normal controls and last follow-up radiographs did not show a significant difference in HEW angle or LPI (*p* = 0.40, *p* = 0.10) (Table 3).

**Table 3:** Data of radiological results comparing among Preoperative, 3 months post-operative, and last follow-up

Radiographic Measurements	Pre operative	3 months post operative	Last follow-up	Normal control	*Preoperative* vs 3 months post operative*	*Preoperative* vs Last follow-up*	3 months post operative vs Last follow-up*	Normal control vs Last follow-up*†*
AP HEW angle (°)	−14.7 ± 6.6	11.5 ± 8.2	12.1 ± 5.2	13.6 ± 5.2	< 0.001	< 0.001	1.000	0.40
AP LPI (%)	− 10.3 ± 5.8	−0.3 ± 5.2	− 0.7 ± 4.6	− 3.7 ± 5.7	< 0.001	< 0.001	1.000	0.10

Values area presented as mean ± standard deviation

*Paired *t*-test

*†*Unpaired *t*-test

### Clinical outcome

The recovery time to the final range of elbow motion in most patients was a mean of 14.3 ± 4.5 weeks (range, 8–24 weeks) postoperatively. Mean ROMs for extension/flexion were − 0.47° ± 5.6° (range, − 15° to 15°) / 130° ± 3.3° (range,120°–140°) preoperatively and 0.6° ± 3.7° (range, 0° to 10°) / 130.3° ± 5.8° (range,125°–140°) at the last follow-up (*p* > 0.05). The motion arc of the elbow at the last follow-up was not significantly different from that at the initial presentation (*p* > 0.05). Meanwhile, the internal rotation angle improved from a mean of 13.5° ± 7.6° (range, 5°–35°) preoperatively to a mean of 3.8° ± 3.7° (range, 0°–10°) postoperatively (*p* <  0.01). When the loss of motion arc was 10° or less after the surgery, it was defined as a successful restoration of elbow motion. All patients achieved successful restoration. Based on the criteria provided by Oppenheim et al. [16], results were rated as excellent for 7, good for 8, and poor for 2 patients. One patient with a poor result had a nonunion. Another patient with a poor result had a transient radial nerve palsy. The mean DASH score was 2.5 ± 3.8 points (range, 0–15 points), which was considered as excellent result. The mean MEPS was 97.0 ± 5.8 points (range, 85–100 points), which was rated as excellent in 14 and good in 3 patients (Table 4).

**Table 4:** Patient data and operative outcomes

Patient number	Time from injury to surgery (years)	Duration of follow up (months)	Humerus-elbow-wrist angle (°)			Lateral prominence index (%)			Internal Rotation (°)		Preoperative range of motion (°)		Postoperative range of Motion (°)		Time to full motion (weeks)	Criteria of Oppenheim et al	Post-operative DASH	Post- operative MEPI
			Pre-operative	Last-Follow-up	Normal	Pre-operative	Last-Follow-up	Normal	Pre-operative	Post- operative	Extension	Flexion	Extension	Flexion				
**1**	**28**	**55**	**−13**	**16.2**	**16**	**−10.4**	**1.5**	**−4.1**	**10**	**0**	**0**	**135**	**0**	**130**	**20**	**Good**	**2.3**	**100**
**2**	**19**	**26**	**−6**	**15**	**23**	**−4**	**−1.4**	**−2.9**	**15**	**5**	**0**	**140**	**0**	**140**	**8**	**Good**	**0**	**100**
**3**	**18**	**24**	**−10**	**5.2**	**13**	**− 18.8**	**−7.3**	**−9**	**10**	**0**	**0**	**130**	**0**	**125**	**20**	**Good**	**6.8**	**85**
**4**	**13**	**40**	**−15**	**10.5**	**20**	**− 14.8**	**−6.3**	**−6.5**	**10**	**5**	**0**	**130**	**0**	**130**	**16**	**Good**	**0**	**100**
**5**	**15**	**28**	**−13**	**18**	**20**	**−10.9**	**6**	**−3**	**5**	**5**	**−8**	**130**	**0**	**130**	**16**	**Excellent**	**15**	**85**
**6**	**10**	**24**	**−6**	**16**	**13**	**−14.7**	**−4.8**	**−8.9**	**5**	**0**	**0**	**130**	**0**	**130**	**16**	**Good**	**0**	**100**
**7**	**10**	**24**	**−12**	**5.2**	**14**	**−3.2**	**−8**	**−12.5**	**10**	**10**	**0**	**130**	**0**	**130**	**12**	**Good**	**0**	**100**
**8**	**12**	**31**	**−14.5**	**9**	**12**	**−3.7**	**−5.9**	**−5.1**	**15**	**0**	**0**	**125**	**0**	**125**	**12**	**Excellent**	**0**	**100**
**9**	**40**	**25**	**−25**	**3.8**	**6**	**−11.8**	**−2.9**	**−9**	**35**	**5**	**0**	**130**	**0**	**130**	**24**	**Poor**	**4.5**	**100**
**10**	**5**	**26**	**−22**	**4.5**	**12**	**1.2**	**4**	**9.1**	**20**	**10**	**0**	**130**	**10**	**130**	**12**	**Good**	**4.5**	**95**
**11**	**4**	**24**	**−14**	**14**	**15**	**−14.2**	**3.7**	**−6.4**	**15**	**0**	**0**	**130**	**0**	**130**	**8**	**Excellent**	**0**	**100**
**12**	**9**	**26**	**−14**	**16.6**	**10**	**−10.6**	**−3**	**−0.5**	**15**	**5**	**0**	**130**	**0**	**130**	**12**	**Good**	**0**	**100**
**13**	**30**	**25**	**−31**	**12.7**	**17**	**−20.1**	**1.5**	**7.5**	**25**	**10**	**0**	**130**	**0**	**130**	**16**	**Excellent**	**0**	**100**
**14**	**8**	**28**	**−12**	**6.5**	**2**	**−14.4**	**−1**	**1.1**	**10**	**0**	**−15**	**130**	**0**	**130**	**8**	**Excellent**	**2.3**	**100**
**15**	**15**	**24**	**−16.5**	**17.4**	**16**	**−4.9**	**4**	**−3.3**	**10**	**5**	**0**	**135**	**0**	**135**	**12**	**Excellent**	**4.5**	**100**
**16**	**16**	**26**	**−17**	**15**	**7.5**	**−10.1**	**3**	**−1.7**	**5**	**0**	**15**	**120**	**0**	**130**	**12**	**Poor**	**2.3**	**85**
**17**	**7**	**24**	**−9**	**20**	**15**	**−9.1**	**5**	**−7.9**	**15**	**5**	**0**	**130**	**0**	**130**	**16**	**Excellent**	**0**	**100**
**Mean**	**15.2**	**28.2**	**−14.7**	**12.1**	**13.6**	**−10.3**	**−0.7**	**−3.7**	**13.5**	**3.8**	**−0.47**	**130.3**	**0.6**	**130.3**	**14.3**		**2.5**	**97.0**

### Complications

Complications arising from primary surgeries included one case of transient radial nerve palsy and one case of delayed union. Patient 15 had high radial nerve palsy after the primary surgery. The radial nerve of the patient showed compression and contusion around the osteotomy site during exploration. This patient recovered within 3 months after the surgery. For the case with delayed union, re-fixation was undertaken using dual plates and concurrent autogenous bone grafting at six months after the initial surgery and successful union was achieved 3 months postoperatively. None of these patients had postoperative infections or late complications such as tardy ulnar nerve palsy, posterolateral rotatory instability, or refracture.

## Discussion

Currently, simple lateral closing wedge osteotomy, step-cut osteotomy, and dome rotational osteotomy are commonly performed for cubitus varus deformity. They can provide satisfactory results in children due to their remodeling capacity and rapid healing ability [5, 6, 11]. However, the distal humerus in adults has large and protrusional condyles. In addition, surgical correction associated with sufficient medial shift of the distal fragment is necessary to achieve satisfactory correction and prevent S-shaped deformity because adults have less healing ability and remodeling capacity than those who are younger [9, 10]. So far, only a few studies have dealt with correction of cubitus varus deformity in adult patients. Labelle et al. [20] have reported that lateral closing wedge osteotomy is difficult to achieve strong internal fixation. In addition, protrusion of the lateral condyle or S-shaped deformity of the elbow may develop postoperatively [20, 21]. Buß et al. [22] have recommended supracondylar humerus closed wedge osteotomy with a locking plate fixation in adults. Moon et al. [21] have suggested medialization of the distal fragment for an effective treatment of cubitus varus deformity with minimized risk of ‘lazy S’ deformity. Oblique closing wedge osteotomy with lateral plating can also be used as a modified procedure to resolve these issues. However, Gong et al. [23] have reported that this technique could result in shortening of the humerus due to larger bone resection than other osteotomies. The simple step-cut osteotomy does not allow translation of the distal fragment after osteotomy. In addition, it induces lateral condylar prominence after correction. This requires a long-term cast immobilization which is not desirable in adults [6]. Dome osteotomy can correct the deformity in coronal and horizontal planes simultaneously with just one bone cut [24]. However, the contracture of the soft tissue around the deformed elbow in adults can often make it difficult to correct the deformity [25].

On the other hand, translation step-cut osteotomy is a simple osteotomy that enables three-dimensional correction of coronal, sagittal, and rotational deformities. The planned osteotomy can be easily performed with the triangular template created before surgery. A triangular wedge-shaped surface created by osteotomy provides firm stability [13]. Y plate provides a sufficiently rigid fixation that permits early active motion and prevents possible complications related to implant failure in cubitus varus deformity of adults [13, 26, 27]. Supplementary bone grafts of excised triangular fragments can also be used to improve bone union. A posterior approach is familiar to elbow surgeons. It has a better surgical field. Supine position is also easy to check whether correction angle achieved by the osteotomy is sufficient or not using an image intensifier with a gross examination.

In the present study, we could correct a mean of 26° of cubitus varus deformity with this osteotomy in adults. The lateral prominence index (LPI) also improved by 9.6% from its preoperative value, showing no significant difference compared to LPI of a normal elbow. None of our patients had lateral prominence after deformity correction. Their post-correction radiological indicators were maintained without significant differences until the final follow-up (Table 3).

Meanwhile, O’Driscoll et al. [3] have suggested that for a tardy posterolateral rotatory instability caused by cubitus varus, osteotomy alone may be adequate if there is only subtle instability or if the patient places only limited demands on the elbow. Carlo et al. [28] have suggested that even high function demand patients (for whom ligament reconstruction is indicated) should be initially treated with a brief period of rehabilitation for at least 3 months. In the present study, although the cubitus varus deformity before surgery had a mean value of 26°, only corrective osteotomy was performed since there was no posterolateral instability.

If a flexion contracture or hyperextension elbow is present, it can be corrected by excising the bone fragment in the posterior part of the V-shaped proximal part during the initial osteotomy. Subsequently, we could correct additional flexion contracture or hyperextension elbow. This study showed good clinical results. All patients had good or excellent results based on DASH and MEPS during the final follow up.

Meanwhile, two cases with poor clinical results were noted based on the Oppenheim criteria. One had a high radial nerve palsy after the primary surgery. Chung et al. [26] have reported a high risk of radial nerve injury with the standard posterior approach during a three-dimensional osteotomy. The radial nerve gives muscular branches to long, medial, and lateral heads of the triceps before lying in a spiral groove on the posterior aspect of the humerus. It then pierces the lateral intermuscular septum to enter the anterior compartment [29, 30]. Uhl et al. [31] have reported that the distance from the articular surface (at the mid-portion or dip of the trochlea) to the radial nerve as it crosses the middle of humerus is 15.8 cm in men and 15.2 cm in women. The mean distance to the point where the radial nerve pierces the septum is 10.0 cm in men and 9.4 cm in women [31]. Carlan et al. [32] have found that the radial nerve is immobilized by obliquely oriented lateral intermuscular septum well distal to its entrance into the anterior compartment. It is known that the radial nerve has very little mobility in this area. Thus, we performed careful dissection to avoid the risk of radial nerve injury during the operation. Nevertheless, radial nerve palsy occurred in one case. Exploration finding showed that the radial nerve was compressed at the anterolateral aspect of the osteotomy site. This might have occurred while medially translating the lateral cortex of the distal fragment after osteotomy. Although iatrogenic injury to peripheral nerves and brachial artery is usually preventable with a posterior approach, careful osteotomy or translation of the distal fragment would be needed due to the possibility of a radial nerve injury.

Another case showed delayed union with screw loosening during the postoperative period. In a previous study of Chung et al. [26], callus was detected at a mean of 4.4 weeks after a three-dimensional corrective osteotomy. Xiao et al. [33] have reported that bone union is achieved for all cases at a mean of 10 weeks after supracondylar closing wedge osteotomy. Lim et al. [15] have reported that osseous union of the closing wedge osteotomy site is obtained for all patients at an average of 17.5 weeks after the operation.

In the present study, all patients except one achieved osseous union of the osteotomy site at a mean of 12.7 weeks. The recovery time to the final range of elbow motion was a mean of 14.3 weeks postoperatively. These clinical results demonstrate that translation step-cut osteotomy using Y plate provides a sufficiently rigid fixation that permits early active motion for adults. Nevertheless, patient 9 had delayed union with screw loosening. Since he had severe deformity compared to a normal elbow, primary surgery was performed with the goal of correction 30° of HEW and internal rotation. The large correction angle inevitably caused a lack of bone contact area during correction. It was also technically demanding to apply a well-contoured plate on the distal humerus. Re-fixation was undertaken using dual plates and concurrent autogenous bone grafting at 6 months after the initial surgery. Other previous reports have shown that osteotomy has similar difficulties due to the lack of bone contact area for severe rotational deformity correction. Complete derotation is difficult to maintain stable fixation stable. It may cause loss of correction [34]. Meanwhile, residual rotation deformity is well tolerated because it is easily compensated by rotation of the shoulder joint. We also agree that complete correction of rotation deformity is not always required. Dual plating can be an alternative choice for osteotomy stabilization in adults requiring large correction angles.

Limitations of this study included its retrospective design, small sample size, short-term follow-up, and the lack of comparative osteotomy groups. The lack of preoperative functional assessment was another limitation of this study. However, the management of distal humeral fracture in children has improved. Cubitus varus deformities are now uncommon in adults. We believe that our study for this deformity in adults will help other surgeons because of its clinical applicability. In future studies, more cases should be assessed and objectivity should be improved by monitoring changes in functional outcomes over time instead of performing one-time assessments.

## Conclusion

Translation step-cut osteotomy using Y plate is an efficient procedure to correct varus alignment and flexion-extension deformities so that they are within normal limits of adults with post-traumatic cubitus varus deformity.

## Data Availability

The datasets used and analysed in this study are available from the corresponding author on reasonable request.

## Abbreviations

AP: Anteroposterior
HEW: Humerus-elbow-wirst
ROM: Range of motion
LPI: Lateral prominence index
ICCs: Intraclass correlation coefficients
CIs: Confidence intervals
DASH: Disabilities of the Arm, Shoulder, and Hand
MEPS: Mayo Elbow Performance Score
